# IL-17 in wound repair: bridging acute and chronic responses

**DOI:** 10.1186/s12964-024-01668-w

**Published:** 2024-05-27

**Authors:** Xingrui Mu, Rifang Gu, Ming Tang, Xingqian Wu, Wenjie He, Xuqiang Nie

**Affiliations:** 1 College of Pharmacy, Zunyi Medical University, Zunyi, 563006 China; 2 Key Lab of the Basic Pharmacology of the Ministry of Education & Joint International Research Laboratory of Ethnomedicine of Ministry of Education, Zunyi Medical University, Zunyi, 563006 China; 3 School Medical Office, Zunyi Medical University, Zunyi, 563006 China; 4 Department of Structural Biology, St. Jude Children’s Research Hospital, Memphis, TN 38105 USA

**Keywords:** IL-17 pathway, Chronic wounds, Inflammation, Diabetic ulcers, Therapeutic targets

## Abstract

Chronic wounds, resulting from persistent inflammation, can trigger a cascade of detrimental effects including exacerbating inflammatory cytokines, compromised blood circulation at the wound site, elevation of white blood cell count, increased reactive oxygen species, and the potential risk of bacterial infection. The interleukin-17 (IL-17) signaling pathway, which plays a crucial role in regulating immune responses, has been identified as a promising target for treating inflammatory skin diseases. This review aims to delve deeper into the potential pathological role and molecular mechanisms of the IL-17 family and its pathways in wound repair. The intricate interactions between IL-17 and other cytokines will be discussed in detail, along with the activation of various signaling pathways, to provide a comprehensive understanding of IL-17’s involvement in chronic wound inflammation and repair.

## Introduction

Skin tissue healing in the human body is highly intricate since it necessitates the coordinated interaction of many cell types in both spatial and temporal dimensions [1]. Specifically, different cells perform distinct functions during hemostasis, inflammation, growth, re-epithelization, and remodeling [2]. Different cells and signal molecules that regulate cell response and dynamic remodeling of the extracellular matrix can repair tissue damage and promote skin wound healing [3]. Wounds are divided into acute wounds (burns and surgical wounds, etc.) and chronic wounds [4]. In the elderly, diabetics, and patients with chronic diseases, the wound usually heals slowly and turns into a chronic wound [5]. To promote wound healing, we must take adequate measures in the pro-inflammatory stage of chronic wounds [6]. Chronic wounds can lead to various issues, including heightened levels of pro-inflammatory cytokines, impaired blood circulation at the wound site in patients, elevated white blood cell count, increased reactive oxygen species, and susceptibility to bacterial infection [7]. Chronic wounds cannot follow and complete the wound-healing process and will develop into chronic ulcers (such as diabetic ulcers), resulting in scars [8]. Currently, due to the aging of the world population and the increasing number of patients with chronic diseases such as diabetes, poor wound healing affects millions of people, and medical and health services are facing a significant challenge [9, 10].

Nearly 20% of all diabetic patients will develop non-healing diabetic foot ulcers (DFUs). DFUs refers to the wound with impaired healing, prolonged inflammation and decreased epithelial kinetics in diabetic patients [11, 12]. DFUs are difficult to heal, and the influencing factors include the production of pro-inflammatory mediators, ischemia caused by microvascular complications, specific metabolic defects, and impaired production of healing-related factors [13]. Therefore, compared with ordinary wounds, DFUs have a longer course of disease and a more complicated mechanism, which has a great impact on the morbidity, mortality and quality of life of patients [14].

The pro-inflammatory cytokine known as interleukin-17 (IL-17) plays a role in the development of a wide range of disorders, including psoriasis, rheumatoid arthritis, and ankylosing spondylitis [15, 16]. In spite of the fact that the pro-inflammatory property of IL-17 is the primary factor in its ability to protect the host, the signal transduction of IL-17 is not constrained in any way [17]. There are connections between the IL-17 signaling pathway and immunopathology, as well as autoimmune disorders and the advancement of cancer [18].

IL-17 was first discovered about 30 years ago. All related receptors of the IL-17 family, consisting of six members (IL-17 A-17 F), have been discovered [19]. At present, studies have shown that IL-17 does not seem to directly act on immune cells, but stimulates stromal cells like endothelial cells, epithelial cells, and fibroblasts, leading to the secretion of various immunomodulatory factors [20]. Fibroblasts, when stimulated by IL-17, can enhance the in vivo proliferation and maturation of immune cells [21]. Within the IL-17 family, both IL-17 A and IL-17 F, alongside IL-17E (commonly referred to as IL-25), have displayed pro-inflammatory properties, which have been extensively studied in experimental and clinical settings [22, 23]. Research has demonstrated that the IL-17 signaling pathway is an essential component in the process of providing targeted treatment for inflammatory skin conditions [24].

New evidence from clinical trials shows that monoclonal antibodies against IL-17 can effectively treat inflammatory skin diseases such as psoriasis, suppurative hidrosis, atopic dermatitis, and pityriasis rubra [24, 25]. Inhibition of IL-17 inflammation can be achieved through direct targeting and indirect targeting [26]. Direct targeting refers to blocking downstream cytokines or targeting their receptors, while indirect targeting refers to blocking upstream cytokines that produce IL-17 [27]. However, the role of the IL-17 family in chronic skin wounds is unclear and a hot research topic in the future [28]. It is necessary to study it further to determine its potential as a therapeutic target for chronic wounds [29]. New research shows that IL-17 A can cause neutrophil inflammation and hinder the process of wound healing [30]. All these shreds of evidence indicate that IL-17 members may be related to impaired skin wound healing. Moreover, studies on the relationship between wound healing and skin tumors confirm how the IL-17 receptor activates the ERK5 axis in Lrig1 stem cells by EGFR and provides insights for proliferation and migration in the process of wound healing and tumor formation [31]. Many studies have emphasized the importance of the IL-17 pathway in promoting wound healing, which provides new possibilities for the treatment and intervention of chronic wounds [32].

In this mini-review, we summarized the IL-17 family as a prospective therapeutic target and discussed the potential pathogenic role and molecular mechanism of the IL-17 family and its route in the process of wound repair.

## IL-17 family

IL-17 is among more than 30 kinds of interleukins found, ranking 17th. When CD4^+^ T cells are activated, they release IL-17, which triggers a cascade of events in various cell types [33]. This includes the production and release of IL-6, IL-8, granulocyte-macrophage stimulating factor (GM-CSF), chemokines, and cell adhesion molecule 1 (CAM-1) by epithelial cells, endothelial cells, and fibroblasts [34]. This ultimately results in inflammation. The cells that produce IL-17 can be roughly classified into two categories [35]. The first category consists of Th17 cells, while the second category comprises innate immune cells produced in peripheral tissues like the skin and the lungs in both humans and mice [36]. These cells include CD8^+^ T cells, natural Th17 cells, natural killer (NK) cells, invariant natural killer T cells (iNKT), and γδ T cells [37].

The IL-17 family comprises six members (IL-17 A-17 F), which have multiple biological functions and can promote immunity to pathogens and drive inflammatory pathology during infection and autoimmunity [38]. The five molecules that belong to the IL-17R family, IL-17RA to IL-17RE, have been recognized as members of the receptor family [23]. It is significant to point out that IL-17 A and IL-17 F are substantially similar to one another and bind to the same receptor [39]. Moreover, both IL-17 A and IL-17 F can be secreted in the form of homodimers or heterodimers connected by disulfide linkages [40]. Although IL-17B, IL-17 C, and IL-17D possess the capacity to elicit inflammatory mediators similar to IL-17 A and IL-17 F, their roles within the immune system remain partially comprehended [41]. It is possible for T cells and innate immune cells to create IL-17E when they are stimulated by an antigen or a pathogen [42]. IL-17E plays a significant role in both the acquired immune responses and the innate immunological responses [43]. According to a number of studies, IL-17E has the potential to cure autoimmune diabetes [44].

### IL-17 pathway

**Fig. 1:**
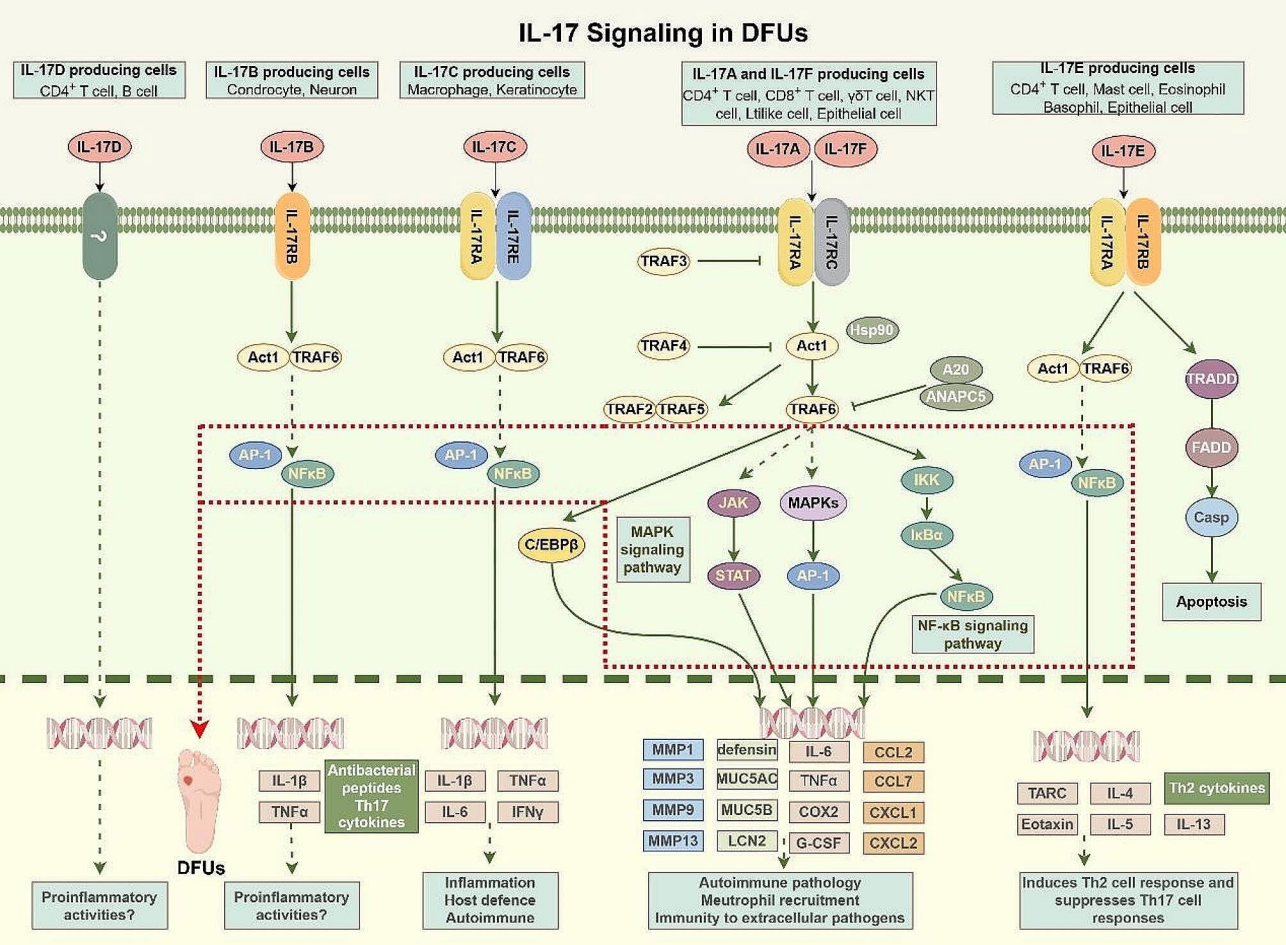
IL-17 Signaling in DFUs. There are six members in the IL-17 family, including IL-17 A (IL-17), IL-17B, IL-17 C, IL-17D, IL-17E (also known as IL-25) and IL-17 F. IL-17 A is the prototype of the IL-17 family. IL-17 F and IL-17 A have the highest homology, about 50%. Both need to bind to the common receptor IL-17RA to start signal transduction. The homology of IL-17B-E and IL-17 A could be better. There are five members in the IL-17 receptor family: IL-17R/IL-17RA, IL-17B R/IL-17RB, IL-17RC, IL-17RD/SEF and IL-17RE. IL-17 family can activate anti-cytokines and chemokines in MAPK, NF-κB, and C/EBPs pathways. Act1 is considered the main mediator in this pathway. (By Figdraw.)

A transmembrane domain is present in every single receptor subunit, as is common knowledge. Nevertheless, the purpose of combining IL-17 A and IL-17RA/RC complex is to recruit ubiquitin ligase Act1 by means of the SEF/IL-17R(SEFIR) domain [45]. Previous research has demonstrated that Act1 has the ability to bind the receptor-related factor 6 (Traf6) of tumor necrosis factor (TNF), which ultimately results in the activation of the nuclear factor κB (NF-κB) and mitogen-activated protein (MAP) kinase pathways (Fig. 1) [46]. Consequently, the activation of these pathways results in the up-regulation or activation of various inflammatory genes, including neutrophil-specific CXC chemokines [47].

IL-17 has been shown to activate NF-κB, a key transcription factor that regulates the expression of various inflammatory cytokines and chemokines [18]. Similarly, the activation of MAPK pathways by IL-17 can lead to enhanced cellular responses that are vital for wound healing processes, such as cell migration and proliferation [48]. Furthermore, the JAK-STAT pathway, when stimulated by IL-17, plays a significant role in immune cell function and inflammation, directly impacting the healing trajectory of DFUs [49–51].

Secukinumab and ixekizumab are monoclonal antibodies that specifically target IL-17 A, a key cytokine involved in the pathogenesis of several autoimmune diseases. Their ability to selectively inhibit IL-17 A has proven beneficial in reducing inflammation and improving clinical outcomes in diseases such as psoriasis and ankylosing spondylitis [52, 53]. Given the similar inflammatory mechanisms that exacerbate DFUs, these inhibitors could potentially modulate the immune response in the wound environment, thereby enhancing healing and reducing complications.

In addition to targeting IL-17 directly, we also explore the potential of targeting downstream effectors such as JAK inhibitors. These inhibitors can modulate the IL-17 pathway indirectly and have shown promise in other inflammatory diseases. JAK inhibitors, like tofacitinib and baricitinib, interfere with the JAK-STAT signaling pathway, which is crucial for the transcription of genes involved in immune and inflammatory responses [54]. By inhibiting this pathway, JAK inhibitors could potentially reduce the excessive inflammatory response observed in DFUs, promoting a more conducive environment for wound healing.

Act1 plays a crucial role in IL-17 signal transduction, and its destruction by the proteasome will occur following the alteration of the ubiquitin chain connected with lysine number 48 [55]. In response to sustained stimulation with IL-17, the F-box E3 ubiquitin ligase, which is a protein that contains β-TrCP, is responsible for catalyzing this process [56]. It is crucial to note that TRAF6 helps activate the MAPK pathway that IL-17 induces to activate transcription factors like AP-1 [57]. Act1 mediates this activation [58]. There are a number of regulatory mechanisms that are used to fine-tune the TRAF6-mediated IL-17 signal transduction in order to reduce the inflammation that is generated by IL-17 [59]. IL-17, despite diminishing the signal output, can still amplify the inflammatory response through a feedforward mechanism [57]. This means that IL-17 has the power to stimulate inflammation further, even though its control over the MAPK and NF-κB pathways is mediated by TRAF6 [60]. This mechanism is involved in the participation of additional transcription factors, such as IkBζ and CCAT/enhancer binding protein (C/EBP).

The interaction between EGFR and the IL-17 receptor complex in skin Lrig1 stem cells is facilitated by TRAF4 upon stimulation with IL-17 [61]. This leads to the close proximity of IL-17R and EGFR, allowing Act1 to recruit c-Src for phosphorylation of EGFR induced by IL-17 A [31]. Consequently, this activation triggers the MEKK3-MEK5-ERK5 pathway [62]. Because of the activation of this axis, Lrig1^+^ cells will be stimulated to create progeny that are responsible for wound healing and carcinogenesis. Certain functions that are derived from the IL-17 pathway are necessary for the healing of wounds. There are multiple sources of IL-17, including CD4^+^ Th17 cells, γδ T17 cells, and CD8^+^ Tc17 cells [63]. According to studies, IL-17 exerts its influence on fibroblasts, causing them to generate vascular endothelial growth factor (VEGF), CXCL1, and REG3α, all of which are crucial for wound healing [64].

On the other hand, this demonstrates that IL-17 is an essential component in the process of facilitating wound repair. VEGF is one of these factors that can stimulate vascular healing following the creation of a wound [65]. On the other hand, CXCL1 has a significant role in the recruitment of neutrophils that secrete MMP-9, which continues to be beneficial for the improvement of wound repair [66]. One of the most critical components in wound repair is that the related cells, which include Lgr5 + stem cells, be present [67]. The synergistic signal transduction of IL-17R and EGFR on these stem cells has been shown to have a major impact on the process of wound repair following an injury, according to the findings of several pieces of research [31]. It is important to note that in addition to secreting IL-17, Tc17 cells are also capable of producing amphiregulin, which is a protein that plays a significant role in wound repair [68].

### The IL-17 pathway regulates skin tissue repair and promotes acute wound healing

Studies conducted in recent years have demonstrated that IL-17 is responsible for driving epithelial HIF-1α [69], which in turn promotes wound repair through glycolysis [70]. IL-17 A/F, which is supplied by skin-dwelling RORγt^+^ γδ T cells that have been expanded, is also required for optimum HIF-1α activation in wound marginal epithelial cells when hypoxia is present [71]. Through the IL-17 A signal transduction of the IL-17RC receptor, it is possible to rapidly stimulate ERK/AKT/mTOR, hence boosting the mRNA and protein levels of HIF-1α [50]. For the purpose of facilitating migration, the IL-17-HIF-1α axis serves as guidance for the transcription and functional program of glycolysis [72]. Based on the findings of this investigation, it has been determined that the IL-17 A-HIF-1α axis has the potential to offer therapeutic prospects for a variety of epithelial inflammation and metastatic disorders [73].

The aseptic skin removal from C57BL/6 mice resulted in an early upregulation of interleukin-1β, TNFα, and oncostatin-M (OSM). However, there was no observed suppression of IL-22 and IL-17 A/F [74]. The introduction of Staphylococcus aureus and Pseudomonas aeruginosa into the wounds not only induced an elevation in IL-1β and OSM expression but also led to a significant augmentation in cutaneous levels of IL-22, IL-17 A, and IL-17 F. In addition, it resulted in an increase in the infiltration of IL-17 A by γδT17 cells and potentially led to the production of IL-22 [75]. Mice with skin infections experienced a deceleration in the wound healing process compared to those without any infection. The combined effects of bacterial infection on IL-22 and IL-17 activity contribute to prolonging the duration required for wound healing.

Moreover, there is an unexpected role played by IL-17 in impeding the recovery of skin wounds. Research has shown the negative impact of IL-17 A on the skin wound healing process during acute wound healing. IL-17 A knockout (KO) mice exhibited enhanced wound closure, myofibroblast differentiation, and collagen deposition while experiencing reduced neutrophil accumulation. This was in comparison to wild-type (WT) animals. On the other hand, the injection of recombinant IL-17 A results in a delay in the healing of wounds, a decrease in collagen deposition, and an increase in the number of neutrophils. In addition, the application of a neutrophil elastase inhibitor to IL-17 A KO mice can enhance wound repair to a level comparable to that observed in WT mice. These findings suggest that IL-17 A acts as a factor impeding the progression of wound healing, and the inflammation caused by IL-17 A-induced neutrophils may contribute to the detrimental effects during skin wound recovery. For the purpose of isolating, identifying, and internalizing ADSC-Exo, fibroblasts (HSF) generated from HS were utilized [76]. When the wound was treated with ADSC-Exo, it healed more quickly, and there was less collagen deposition than in the other groups. Another aspect to consider is the strong expression of miR-192-5p in ADSC-Exo and ADSC-Exosomal miR-192-5p, which holds the potential for improving hypertrophic scar fibrosis. Simultaneously, miR-192-5p targets the expression of IL-17RA, leading to a reduction in the availability of pro-fibrotic protein.

Furthermore, it was observed that the expression of IL-17RA was elevated in both HS and HSFs. Silencing IL-17RA led to a reduction in the levels of Col1, Col3, α-SMA, and p-Smad2/p-Smad3 in HSFs while simultaneously inducing an upregulation of SIP1 expression. One of the most notable benefits of inhibiting IL-17RA is that it speeds up the healing process of wounds, reduces the formation of collagen, and controls the Smad pathway in HSFs.

MAIT cells in the skin are the primary group of lymphocytes that produce IL-17 A in adults [77]. These cells exhibit evident transcriptional characteristics and are able to react to the symbiotic link between the skin and the immune system in a manner that is dependent on IL-1, IL-18, and antigen factors. Therefore, the local activation of MAIT cells in the skin is beneficial to the healing process of wounds.

Antibacterial and antioxidant activities are associated with Thymol (THY) [78]. More recently, studies have demonstrated that THY possesses both anti-inflammatory and therapeutic effects. Based on in vivo investigations, significant differences were observed in the levels of IL-1, IL-17, TNF-α, AST, MPO, and CRP between the experimental group and control group. The control group exhibited higher levels compared to the experimental group. THY possesses several important properties, such as anti-inflammatory effects and potential improvements in digestive system function, cardiovascular health, respiratory system function, skin damage repair, and burn healing. It is essential to investigate and elucidate the dose-response relationship as well as the mechanism of action of THY, particularly in the context of the utilization of THY as a therapeutic agent.

Several studies have delved into the impact of dendritic epidermal T cells (DETCs) and Vγ4 T lymphocytes on mouse epidermal cell proliferation, differentiation, and wound healing [79]. In relation to the wound healing process, DENTC’s secretion of IGF-I can promote the growth of mouse keratin 14 positive epidermal cells and hinder their final maturation. Conversely, Vγ4 T lymphocytes’ secretion of IL-17 A can stimulate the proliferation and final maturation of mouse keratin 14 positive epidermal cells. Consequently, both IGF-I and IL-17 A possess the potential to impact wound healing.

Research was conducted to investigate the site-specific modulation of inflammatory mediators in order to enhance the healing of various wounds in lizard tails and limbs [80]. This was accomplished by analyzing the level of healing that occurred in various wounds on the lizard’s tail and limbs. The results of this study shed light on the new function that IL-17 and IL-22 play in wound healing. The reduction of IL-17 in the tail leads to the emergence of IL-22, which promotes wound healing without scar formation by creating a favorable environment for repair. On the other hand, the synergistic rise of IL-17 and IL-22 creates a niche that is suited for the healing of limb scar wounds, which eliminates the potential for renewal of the scar.

The potential for healing that cinnamaldehyde possesses in mouse skin wounds that have been infected with Pseudomonas aeruginosa was investigated, as was the mechanism that is involved in this reaction [81]. Additionally, the metabolic rate of Pseudomonas aeruginosa was slowed down by the sub-inhibitory dose of cinnamaldehyde, which also reduced the bacteria’s capacity to form biofilm and cause hemolysis. By applying cinnamaldehyde to the skin wound that is infected with Pseudomonas aeruginosa on a daily basis, it is possible to lessen the number of bacteria that are present in the tissue and to hasten the healing process. Cinnamaldehyde was used to treat wound samples, and the results showed that low amounts of VEGF, nitric oxide, and IL-17 were determined. Anchin 1, which is the pharmacological target of cinnamaldehyde, was able to block the transient receptor potential, abolish its healing function, and partially reverse the inhibitory effect that the substance had on VEGF and IL-17. When it comes to improving the healing process of skin wounds that have been infected by Pseudomonas aeruginosa, it is believed that the local application of cinnamaldehyde at a concentration that is below the inhibitory threshold could be an intriguing strategy.

## Targeting IL-17 pathways for enhanced healing in chronic wounds and DUFs

### ECM formation and remodeling

In diabetic conditions, the extracellular matrix (ECM) undergoes significant alterations that critically impair wound healing processes. This section delves into the modifications of ECM components under diabetic states and their implications for wound repair. Diabetic environments are characterized by persistent hyperglycemia, which influences the structure and function of ECM proteins such as fibronectin and collagen. These proteins are essential for the structural integrity and signaling functions necessary for effective wound healing.

IL-17 plays a pivotal role in the dysregulation of ECM remodeling in DFUs. The cytokine influences various cellular activities that disrupt normal ECM composition and organization. For instance, IL-17 has been shown to enhance the expression of matrix metalloproteinases (MMPs), which are enzymes that degrade ECM components, leading to an imbalance in the synthesis and degradation of fibronectin and collagen [28]. This disruption contributes to the chronicity of wounds observed in diabetic patients, as the ECM fails to provide a conducive scaffold for cell migration and proliferation, essential for wound closure.

Furthermore, IL-17 can induce the expression of pro-inflammatory cytokines in DFUs, exacerbating inflammation and further hindering the healing process by altering ECM dynamics [28]. The chronic inflammatory state maintained by IL-17 not only prevents the resolution of inflammation but also promotes fibrosis, which can lead to the stiffening of the ECM and impaired functionality [82].

**Fig. 2:**
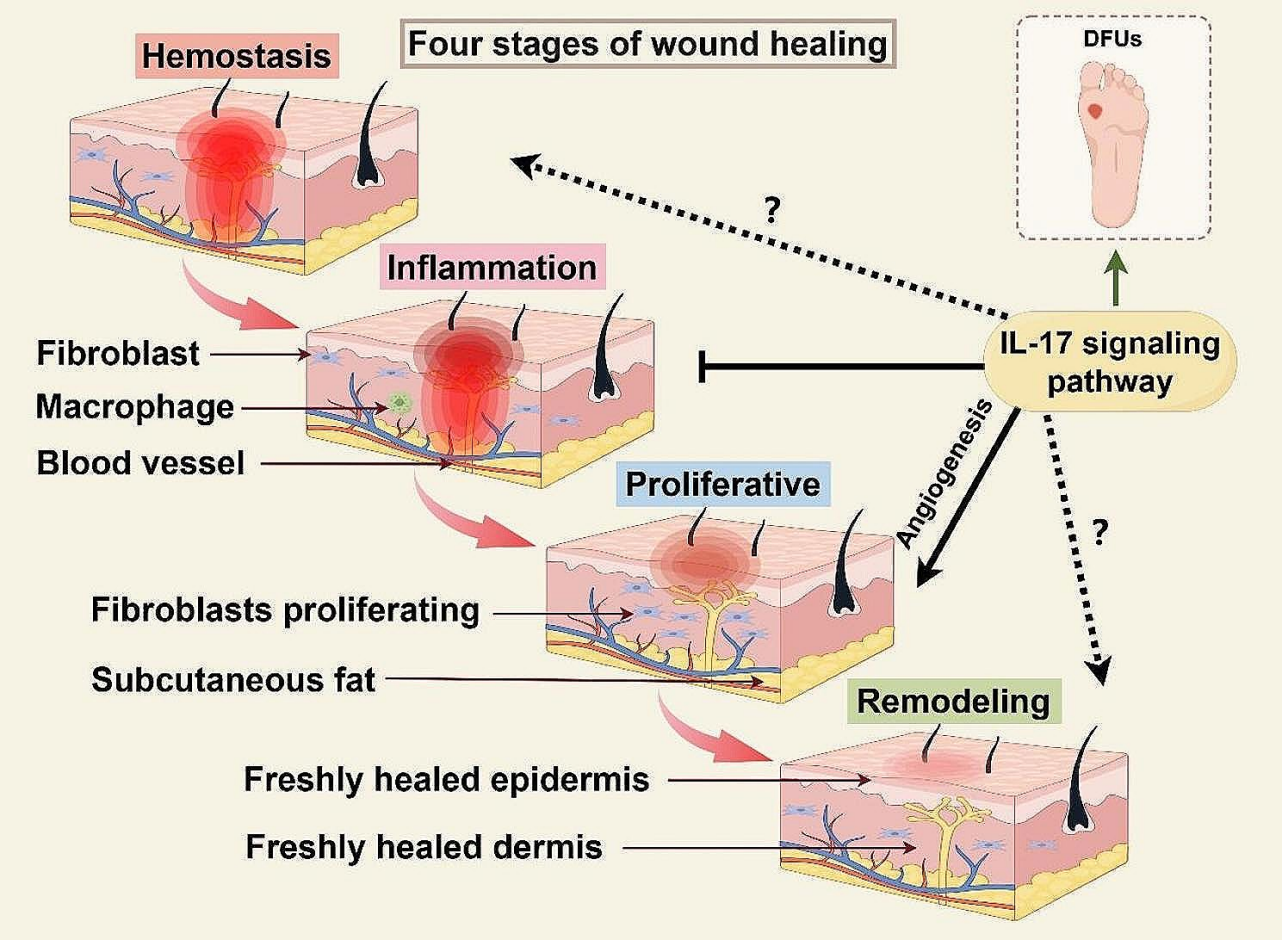
The role of IL-17 pathway in four stages of chronic wound healing. Wound healing is divided into four stages: (1) The first stage of healing is hemostasis. At this stage, the body activates the emergency repair system and blood coagulation system. (2) The second stage is called the defense/inflammation stage. The emphasis is on destroying bacteria and removing debris, which is often accompanied by edema, erythema (red skin), heat and pain. (3) The third stage is the proliferation stage. This period can be divided into epithelial regeneration and granulation. It is mainly the proliferation and differentiation of fibroblasts and endothelial cells and the formation of new capillaries, which together form granulation tissue and fill and cover the wound. (4) The fourth stage is the remodeling stage. After the repair stage, the wound has initially healed. With the passage of time, the scar tissue, scab, etc. of the wound are gradually adjusted to repair the tissue to adapt to the physiological function, and finally the appearance and function of the injured part are improved. (By Figdraw.)

Understanding the interaction between IL-17 and ECM components in DFUs is crucial for developing targeted therapies that can modulate this pathway, potentially reversing the impaired healing processes seen in diabetic patients (Fig. 2).

### Angiogenesis

IL-17 is known to exert a dual influence on angiogenesis, acting as both a promoter and an inhibitor of new blood vessel formation, depending on the cellular and cytokine milieu. Research indicates that IL-17 can enhance the expression of angiogenic factors such as VEGF, which stimulates endothelial cell proliferation and new vessel formation. However, IL-17 also contributes to an inflammatory environment that can adversely affect endothelial cell function and angiogenesis. For instance, IL-17 has been shown to increase the production of pro-inflammatory cytokines and chemokines, which can lead to endothelial dysfunction and impaired angiogenic response [83, 84].

The complexity of IL-17’s role in angiogenesis under diabetic conditions suggests that targeting this cytokine could offer a nuanced approach to modulating angiogenic responses in DFUs. By either enhancing its angiogenic promotions or mitigating its inhibitory effects, therapeutic strategies could be tailored to the specific needs of the wound healing stage and individual patient pathology.

### Collagen formation

Collagen, a principal component of the extracellular matrix, plays a crucial role in wound healing by providing structural support and integrity. In the context of DFUs, efficient collagen synthesis and stabilization are often compromised, contributing to the chronic nature of these wounds.IL-17 has been shown to induce the expression of matrix metalloproteinases (MMPs), particularly MMP-1 and MMP-9, which degrade collagen and other ECM components. This enzymatic activity disrupts the normal collagen architecture, leading to a weakened structural matrix in the wound bed and impaired healing outcomes [85, 86]. Additionally, IL-17 can skew the balance of collagen synthesis by fibroblasts, promoting the production of non-functional, disorganized collagen fibers, further exacerbating the healing challenges in DFUs [87].

The destabilizing effects of IL-17 on the collagen matrix not only delay wound closure but also affect the mechanical properties of the healed tissue, increasing the susceptibility to re-injury. By elucidating the mechanisms through which IL-17 modulates collagen dynamics, our manuscript underscores the potential therapeutic benefits of targeting this cytokine to improve wound healing in diabetic patients.

### Infection

IL-17 is known to play a pivotal role in the host defense against pathogens by regulating the activities of various immune cells and the production of antimicrobial peptides. However, in the context of DFUs, the dysregulated expression of IL-17 can contribute to an exacerbated inflammatory response and altered wound microbiome, which can complicate infection management and impair healing processes.

For instance, elevated levels of IL-17 can enhance the recruitment of neutrophils and other immune cells to the wound site, which, while crucial for combating infections, can also lead to tissue damage if not properly regulated. Additionally, IL-17 can affect the expression of antimicrobial peptides that are essential for controlling microbial growth directly at the wound site [88–90]. This provides a clearer link between infection dynamics and other pathological aspects of DFUs, such as inflammation and tissue remodeling, emphasizing the potential therapeutic benefits of modulating IL-17 to improve outcomes in DFUs management.

### Inflammation

Even though diabetic ulcers are a formidable complication of diabetes, the therapeutic methods that are currently available are unable to generate satisfying results. Through the use of the RNA-seq technique, the effects of Cortex Phellodendri Liniment (HB) and berberine on the wound healing process in diabetic rats that a high-fat diet and streptozotocin injection had caused were investigated further [91]. When locally applied, HB exhibits the potential to enhance wound healing in individuals with diabetes, including diabetic rats. Additionally, it exerts an impact on various mechanisms, notably the IL-17 signaling pathway, which holds particular importance. HB effectively reduced the excessive expression of IL-17 and its downstream targets (such as CXCL1, CCL2, MMP3, MMP9, G-CSF, IL-1β, and IL-6) in DFUs while simultaneously enhancing T-AOC levels, SOD activity, and GSH levels.

Furthermore, IL-17 A was inhibited by its inhibitors or antibodies, which caused a significant increase in the rate of wound healing. The levels of nitro tyrosine and 8-OHdG are decreased, while the expression of CD31, PDGF-BB, and ANG1 that are associated with angiogenesis is increased; the amount of cleaved caspase-3 is inhibited, and TIMP1 and TGF-β1 are promoted. Additionally, berberine, which is the primary component of HB, suppresses the IL-17 signaling pathway and accelerates the healing of DFUs.

Research has shown that the improved GO-based wound dressing has the potential to increase the production of sEVs by increasing the amount of miR-21 that is produced by AD-MSCs [92]. In order to enhance wound healing in the diabetic foot (DF), it has been discovered through bioinformatics research and testing that PVT1 is the most important long noncoding RNA (lncRNA). Additionally, the axis of PVT1/PTEN/IL-17 is altered through the modification of miR-21. The GSK-3b level can be reversed by the PI3K/Akt signaling pathway, counteracting the impact of IL-17. At the same time, it can also ensure proper checkpoint angiogenesis and improve the damaged microcirculation under DF background conditions. This approach is employed to provide additional insights into the role of materials in controlling the recovery process of diabetic ulcers.

Interleukin-25 (IL-25) is a type of protein in the body known as a cytokine. Its primary role is to act as an alarm system and respond to any damage that occurs to tissues [93]. IL-25, in addition to its role in tissue regeneration and glucose homeostasis maintenance, also plays a crucial yet incompletely understood function in the healing process of DFUs. Studies have revealed that interleukin-17 receptor B (IL-17RB) acts as a functional receptor for IL-25. Interestingly, the expression of IL-17RB is significantly suppressed in the injured skin of diabetic patients suffering from DFUs and in mice with streptozotocin (STZ)-induced diabetes [94]. The localized application of recombinant IL-25 protein may potentially to improve angiogenesis and collagen deposition in DFUs beds, thereby optimizing wound healing delay.

Additionally, exogenous IL-25 can safeguard endothelial cells against the negative effects of high glucose levels on cell migration and tube formation in vitro. In DFUs, IL-25 is responsible for increasing the expression of endothelium-specific CD31. Furthermore, it was observed that IL-25 mediated the signal transduction of IL-17RB, thereby inhibiting the suppression of the Wnt/β-Catenin pathway in both in vivo and in vitro experiments conducted on HUVECs from diabetic mice. Moreover, it triggered the activation of AKT and ERK 1/2 in HUVECs upon exposure to elevated glucose levels. This investigation aimed to ascertain the beneficial regulatory influence exerted by IL-25-induced IL-17RB signal transduction on the recovery mechanism of wounds in individuals with diabetes. The outcomes of their study implied that the induction of IL-25-induced IL-17RB signal transduction might represent a promising innovative approach to rectifying insufficient healing of wounds in diabetic patients. To investigate the differences in the innate immune response that occur throughout the healing process between DFUs and normal wounds, a mouse model was utilized. On the back skin of BKS, two full-thickness wounds of 5 millimeters each were generated. The purpose of this study is to establish whether or not DFUs contain higher amounts of IL-17 and IL-20 [95]. These cytokines are also raised in inflammatory skin illnesses like psoriasis, and it is possible that they could be therapeutic targets that could assist in the healing of diabetic infections and wounds.

PZH, also referred to as Pien Tze Huang, is an approved medication utilized for the standardized and validated treatment of diverse wound types. The primary objective of this research was to systematically examine the impact and mechanism of administering PZH through intragastric injection (I-PZH) on the wound healing process in individuals with diabetes [96]. However, it does not have any effect on the level of glucose in the blood when the rats are fasting. I-PZH can stimulate wound healing, promote the synthesis of extracellular matrix, and maintain the weight of rats. The RNA-seq analysis revealed that I-PZH exhibited anti-inflammatory properties, with TLR2, IL-17 A, and IL-1β identified as the most significant common targets. Further investigations demonstrated that the application of I-PZH in DFUs resulted in decreased levels of TLR2, IL-17 A, and IL-1β, while also promoting the healing process of DFUs. When inflammation occurs, myeloid-derived suppressor cells (MDSC) begin to collect. These cells regulate Kruppel-like factor 4 (KLF4), which in turn promotes the healing of chronic wounds. The aim of this study is to explore the potential contributions of MDSC and KLF4 in the recovery process of wounds in individuals with diabetes. The wound healing process was evaluated using a pressure ulcer (PU) model derived from an *ob*/*ob* mouse. The absence of KLF4 in the diabetic PU model led to a reduction in the formation of mesenchymal stem cells (MSCs), an increase in the proliferation of Th17 cells, and a noticeable delay in the restoration of wound healing.

On the other hand, APTO-253 is responsible for activating KLF4, which speeds up the healing process of wounds. Additionally, there was an increase in the population of MDSC cells alongside a decrease in the number of Th17 cells [97]. There is evidence suggesting that MDSCs have the potential to influence the differentiation of Th17 cells by utilizing cytokines. According to the findings of our in vitro research, an increase in the expression of KLF4 in MDSCs results in a reduction in the number of Th17 cells, which in turn leads to a drop in the number of cytokines that are required for the differentiation of Th17 cells.

It is generally accepted that various subsets of macrophages have distinct impacts on wound healing, which can sometimes be in direct opposition [98]. Using multi-label flow cytometry and RNA expression array analysis, it is characterized by low Ly6c and high MHCII levels in wound granulation tissue. This subset experienced a proportional and absolute increase throughout the normal wound-healing process. However, it was absent in the *ob*/*ob* and MYD88^−/−^ models, which exhibit delayed healing. It was also shown that IL-17 is the primary cytokine that differentiates this population from pro-inflammatory macrophages. Furthermore, it was demonstrated that reducing IL-17 by blocking Ab or IL-17 A^−/−^mice could speed up both normal and delayed healing processes.

In the intricate environment of DFUs healing, IL-17-secreting immune cells play a multifaceted role. While they contribute to the inflammatory processes that exacerbate wound pathology, they are also pivotal in combating infection and facilitating the tissue repair mechanisms. Recent studies suggest that the timing and intensity of IL-17 expression are critical, with early-phase activity linked to necessary inflammatory responses and prolonged activity associated with chronic inflammation and delayed healing [99]. Thus, therapeutic strategies that selectively modulate IL-17 production or activity during specific phases of the healing process may offer a more balanced approach to managing DFUs inflammation without compromising tissue repair and regeneration [100].

For instance, localized therapies that reduce IL-17 activity during the inflammatory phase of wound healing but taper off as healing progresses could potentially optimize outcomes. Such approaches would benefit from advanced drug delivery systems that allow for controlled release of IL-17 inhibitors, aligning drug activity with the wound healing stages [101].

## Conclusion

**Fig. 3:**
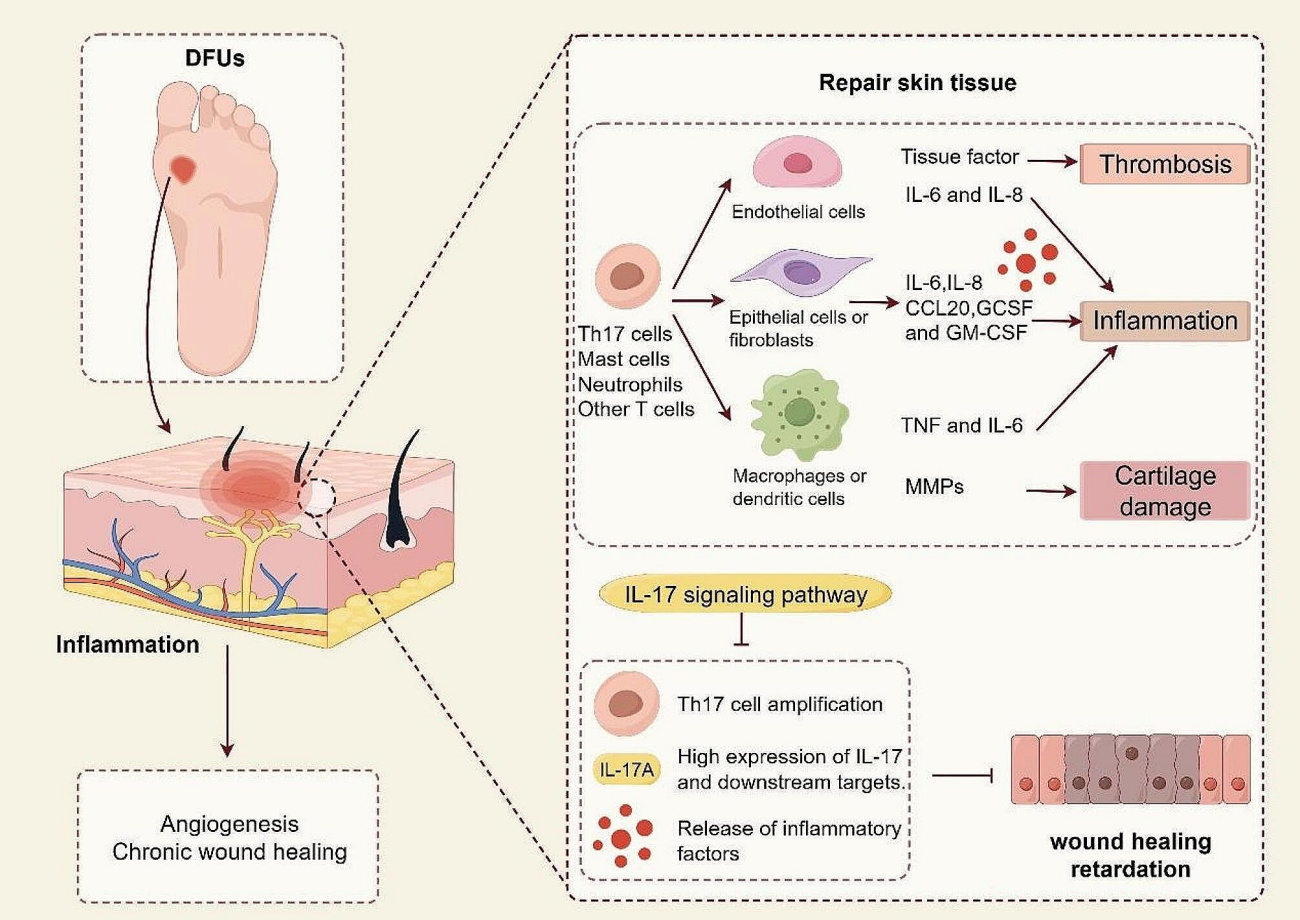
The process of IL-17 signaling pathway promoting chronic wound healing. In the process of chronic wound healing of skin tissue, the synergistic effect of endothelial cells, fibroblasts, macrophages, and other cells is inseparable. Effective regulation of the IL-17 signaling pathway can reduce Th17 cell proliferation, control the high expression of downstream targets, and reduce the release of inflammatory factors. In order to improve the delay of wound healing, regulating the IL-17 signaling pathway may be a new effective strategy. (By Figdraw.)

The involvement of IL-17 family members in dermatological conditions suggests that they may play a significant role in developing and progressing of chronic wounds. Chronic wounds, such as pressure ulcers, diabetic foot ulcers, and venous leg ulcers, are characterized by impaired healing processes and prolonged inflammation. Understanding the contribution of IL-17 family members to chronic wound formation could lead to novel therapeutic strategies targeting this pathway. By modulating IL-17 levels or blocking its signaling pathways, it might be possible to alleviate chronic inflammation and promote more efficient wound healing. An essential goal in the future is to understand the precise mechanism of the IL-17 pathway and TH17 cell regulation during inflammation and wound healing to design targeted therapy for inflammatory diseases, including infection and immune mediation. Overall, further research into the involvement of IL-17 family members in dermatological conditions holds promise for improving our understanding of chronic wound pathogenesis and developing targeted interventions for better patient outcomes (Fig. 3).

## Data Availability

No datasets were generated or analysed during the current study.

## Abbreviations

Act1: Nuclear factor-kappa B activator 1, or TRAF3IP2, or CIKS
AKT: Serine/threonine kinase 1
ANG1: Angiopoietin-1
CD31: Platelet endothelial cell adhesion molecule-1
CXCL1: Chemokine (C-X-C motif) ligand 1
DFUs: Diabetic foot ulcer
ECM: Extracellular matrix
EGFR: Epidermal growth factor receptor
ERK: Extracellular regulated protein kinases
HIF-1: Hypoxia inducible factor-1
HSF: Human skin fibroblasts
KLF4: Krueppel-like factor 4
Lrig1: Recombinant leucine rich repeats and immunoglobulin like domains protein 1
MDSC: Myeloid-derived suppressor cells
MHCII: Major histocompatibility complex class ii
MMP3: Matrix metalloproteinase-3
MHCII: Major histocompatibility complex class ii
NF-κB: Nuclear factor kappa-B
PDGF-BB: Platelet-derived growth factor BB
Smad: Drosophila mothers against decapentaplegic
Th17: T helper cell 17
TIMP1: Tissue Inhibitor of Metalloproteinase 1
TLR2: Toll-like receptor 2
TRAF6: TNF receptor associated factor 6
VEGF: Vascular endothelial growth factor
